# Single-Bit, Self-Powered Digital Counter Using a Wiegand Sensor for Rotary Applications

**DOI:** 10.3390/s20143840

**Published:** 2020-07-09

**Authors:** Janki Chotai, Manish Thakker, Yasushi Takemura

**Affiliations:** 1 Instrumentation and Control Engineering Department, Gujarat Technological University, Ahmedabad 382424, Gujarat, India; 2 Instrumentation and Control Engineering Department, L D. College of Engineering, Ahmedabad 380015, Gujarat, India; dr.mtthakker@ldce.ac.in; 3 Electrical and Computer Engineering, Yokohama National University, Yokohama 240-8501, Japan; takemura-yasushi-nx@ynu.ac.jp

**Keywords:** Wiegand sensor, rotary applications, energy harvesting, Internet of Things (IoT), wireless sensor network (WSN)

## Abstract

This work explores energy harvesting from rotary motion using a Wiegand sensor, which is a magnetic sensor that induces a voltage pulse when the magnetization is reversed. The main feature of the Wiegand sensor is that a pulse is generated regardless of how slowly magnetism reversal occurs. Self-sustained sensors play major roles in advancing the Internet of Things (IoT) and wireless sensor networks (WSN). In this study, we identified a linear relationship between rotational motion, magnetic field reversal, and the rotational frequency generated by the Wiegand sensor. In addition, the maximum energy per pulse and its dependence were derived analytically. A maximum energy of 130 nJ per pulse was reported for the sensor used. We developed a single-bit, self-powered digital counter that was sufficiently driven with 38 nJ of energy. In this study, single rotations were measured without the need for external power.

## 1. Introduction

The Internet of Things (IoT) has been rapidly gaining ground in recent times. The IoT is a wide range of sensors and sensor systems that communicate through wireless sensor networks (WSN) via the internet in a variety of application environments and are generally battery operated. Replacing battery-based sensors with self-powered sensors can reduce maintenance issues; therefore, energy harvesting has become a solution for the operation of devices without batteries [1]. The IoT trend has created an IoT subset known as the Industrial Internet of Things (IIoT) or Industry 4.0 [2], and energy harvesting from industrial environments for IIoT platforms has also become a recent trend [3,4,5]. Similarly, rotational motion already exists in many industrial motion-control and flow-metering applications. Due to its continuous and predictive output, energy scavenging from rotary motion is considered to be a promising research area. A piezoelectric transduction mechanism has been used to harvest this rotational energy [6,7,8], and various types of electromagnetic-mechanism-based energy harvesters have been discussed [9,10,11]. One of the major energy-harvesting constraints from rotary motion is the detection and generation of power at extremely low rotational speeds.

In this study, we used a Wiegand sensor for such applications. A Wiegand sensor is a magnetic sensor that induces a voltage pulse when magnetization is reversed. Magnetization reversal in magnetic wires with bistable magnetization states is characterized by a large Barkhausen jump, which is commonly known as the Wiegand effect [12,13]. The Wiegand sensor harvests significantly less energy in comparison to the other mentioned techniques. However, the main advantage of the Wiegand sensor is that the amplitude and width of the induced voltage pulse are constant for an extremely slow-moving rotating device, and the energy generated by a single pulse is sufficient to perform simple arithmetic operations and store the results in nonvolatile memory. In a recent study [14], a Hall sensor was operated by the single pulse generated by a Wiegand sensor, and ~600 nJ of energy was obtained from a single voltage pulse. Saggini et al. [15] proposed low-power energy harvesting solutions using a Wiegand sensor in a one-shot manner and with battery charging options. Integrated circuits powered by energy harvesting from the Wiegand effect are commercially available for multiturn counters/encoders [16]. Consequently, the results published from recent research studies have opened interesting research avenues.

In this work, the relationship between rotational speed, the frequency generated by the sensor, and the number of magnetic field reversals was derived. We subsequently used an equivalent electrical model of a Wiegand sensor, derived the optimal conditions for energy per pulse, and developed a single-bit, self-powered digital counter circuit with an active rectifier. With the developed circuit, single revolutions (i.e., extremely low-frequency signals) were measured, highlighting the potential of Wiegand sensors in rotary applications.

## 2. Energy Harvesting from a Wiegand Sensor

### 2.1. Experimental Setup

**Figure 1:**
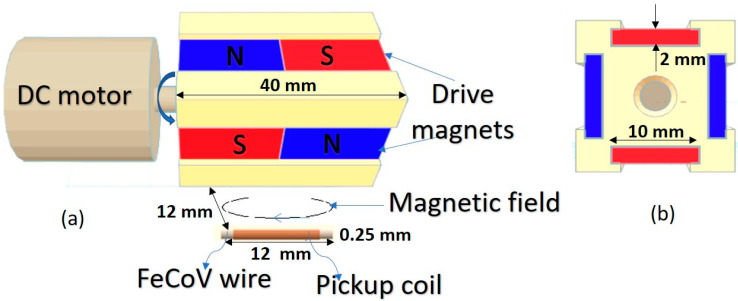
A Weigand sensor (FeCoV wire and pickup coil) and the arrangement used ((**a**) front view and (**b**) side view) to generate electrical power.

**Figure 2:**
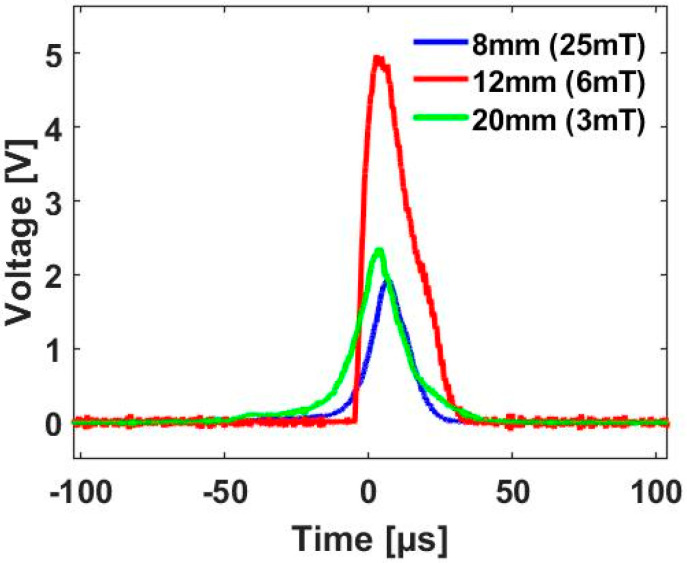
Voltage pulses at distances of 8, 12, and 20 mm corresponding to magnetic field intensities (µ_0_*H*) of 25, 6, and 3 mT.

The Wiegand sensor used in this study was composed of a twisted FeCoV (Fe_0.4_Co_0.5_V_0.1_) wire and a detection coil with 2000 turns. The length and diameter of the wire were 12 and 0.25 mm, respectively. When torsion stress was applied to the wire, the outer shell near the surface became magnetically soft. When the stress was released, two layers appeared: the outer layer, commonly referred to as the soft layer, which generated a coercive force of µ_0_*H* = 2 mT, and the inner layer, commonly referred to as the hardcore, which generated a coercive force of µ_0_*H* = 8 mT. This magnetic wire exhibited uniaxial magnetic anisotropy along its length. The magnetization properties of the wire were reported in detail in a previous publication [14]. The internal resistance *R*_w_ across the pickup coil was measured to be 170 Ω; the internal inductance *L*_w_ of the pickup coil was 2.5 mH, as measured with an LCR meter. Alternating magnetic excitation was provided by a pair of NdFeB magnets (sizes of 20 × 10 × 2 mm^3^) attached to the shaft of the direct current (DC) motor, as shown in Figure 1. Figure 2 shows a typical waveform of the output voltage pulse induced in the pickup coil when an external alternating magnetic field was applied by the magnets, as indicated in Figure 1. Output pulse voltage is clearly a function of the magnetic flux intensity at particular distances from the pickup coil. A magnetic field of µ_0_*H* = 6 mT is sufficient to generate an output pulse *V*_w_ ≈ 5 V and a pulse width *T*_w_ ≈ 40 µs. The amplitude of the output voltage pulse *V*_w_ increases with excitation frequency, but the full pulse width *T*_w_ is independent on the drive field intensity [17]. The results in Figure 2 were obtained with rotational frequencies adjusted such that they were lower than 1 Hz.

### 2.2. Relationship between Rotational Frequency and Rotational Speed

The frequency of the alternating signal generated across the terminals of the Wiegand sensor is denoted as the rotational frequency f and can be varied by varying the speed *S* of the DC motor. In turn, the speed of the DC motor and rotational frequency exhibit a linear relationship. The rotational frequency can be altered by altering the number of magnetic field reversals *N* while keeping the speed constant. Thus, the linear relationship between the rotational frequency, rotational speed, and the number of field reversals, is represented as

(1) $$f=\frac{N\cdot S}{120}$$

where *f* is the rotational frequency in Hz, *S* is the rotational speed in revolutions per minute (rpm), and *N* is the number of field reversals. Thus, the rotational frequency is directly proportional to the number of field reversals and the speed of the rotations.

### 2.3. Maximum Energy per Pulse

**Figure 3:**
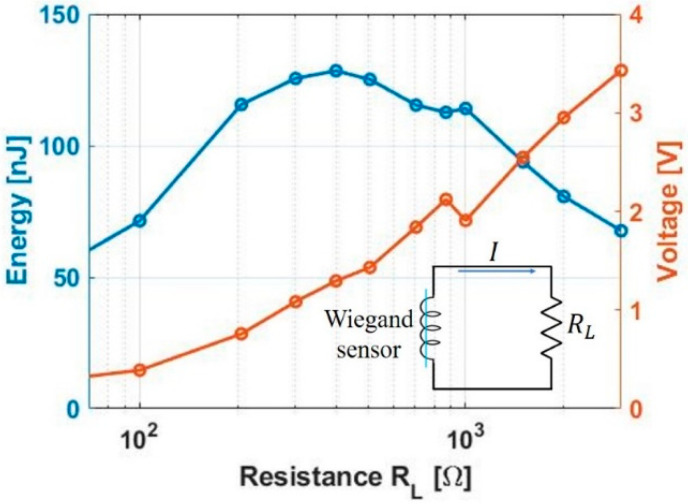
Energy consumption in the load resistor *R*_L_ from a single Wiegand voltage pulse and corresponding voltage drop.

Energy generation using a single Wiegand voltage pulse is shown in Figure 3, in which the load resistor *R*_L_ is connected in series with the Wiegand sensor for extremely low rotational frequencies. The voltage drop across the load resistor *R*_L_ is plotted by varying the value of *R*_L_ (Figure 3). The energy consumed in resistor *R*_L_ is calculated by the time integral of *I*^2^*R*_L_ for each pulse, and the maximum energy consumption reported is 130 nJ for *R*_L_ = 400 Ω.

**Figure 4:**
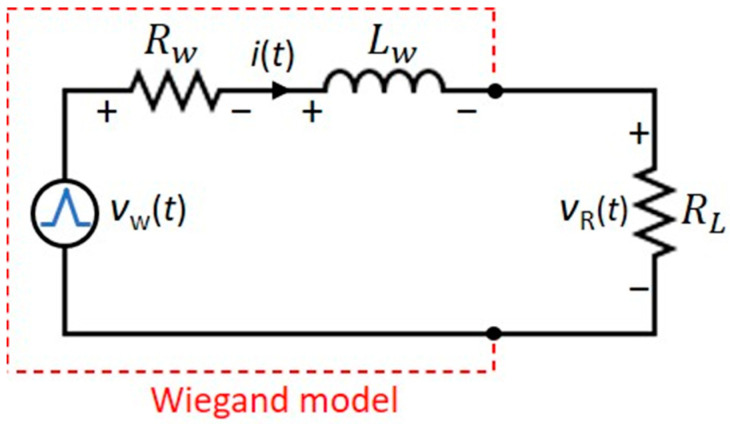
Equivalent electrical model of the Wiegand sensor.

A simplified electrical model of the Wiegand sensor has been discussed [18,19], with the Wiegand pulse approximated by a triangular pulse, as shown in Figure 4.

According to the maximum power-transfer theorem for alternating sources, the maximum power is transferred to the resistive load when the load resistance is equal to the equivalent impedance of a given network, viewed from the load terminals. In this analysis, we considered the effects of both *R*_w_ and *L*_w_. The equivalent impedance of the Wiegand sensor is denoted as *Z_w_* = *R_w_* + j*X_Lw_*. So, for maximum power transfer,

(2) $$\;\;R_{L}=\sqrt{R_{w}{}^{2}+X_{L}{{}_{w}}^{2}}$$

where *R_w_* is the internal resistance and inductive reactance *X_Lw_* = 2π*L*_w_/*T*_w_. From the experimental results shown in Figure 3, *v_R_*(*t*) drops to 1.3 V for *R_L_* = 400 Ω, which is approximately one-fourth of the peak voltage *V_w_*. Considering that

(3) $$v_{R}\left(t\right)=\frac{v_{w}\left(t\right)}{4}$$

the maximum energy per pulse available from the sensor can be derived analytically by integrating power over the period of *T_w_:*

(4) $$E_{w\;max}=\int_{0}^{T_{w}}\frac{v_{w}{}^{2}\left(t\right)}{16Z_{w}}dt=\frac{V_{w}{}^{2}}{16Z_{w}}T_{w}\;$$

Substituting values into Equation (4) leads to *E_w max_* ≈ 150 nJ; thus, the obtained analytical value is close to the experimental result. However, it should be noted that the internal inductance *L*_w_ is a function of the externally applied, time-varying magnetic field, as discussed by Takahashi et al. [19].

## 3. Single-Bit, Self-Powered Digital Counter Design

**Figure 5:**
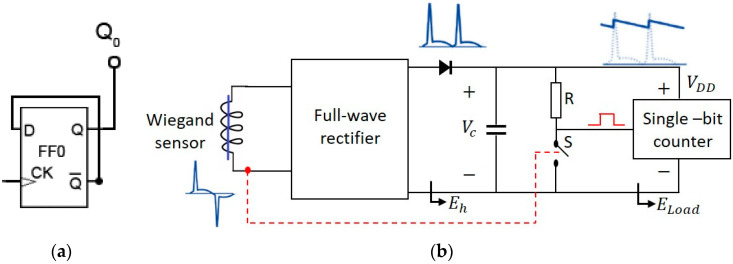
(**a**) Single-bit asynchronous counter using a D flip-flop. (**b**) Power supply plus clock signal generation for the asynchronous counter.

Many motion control and flow metering applications are required to measure the speed of a rotor or turbine. In such cases, the Wiegand sensor may be extremely useful as a self-powered sensor. The rotational frequency and speed of the motor are linearly proportional for a Wiegand sensor with a single-bit asynchronous D flip-flop counter circuit (CD4031B), as shown in Figure 5a; this sensor was operated without an external power supply using the proposed scheme. In this research, a simple capacitor-based power supply architecture was used to operate the D flip-flop. Thus, the alternating signal generated by the Wiegand sensor was converted into a DC signal with a full-wave bridge rectifier that charged the storage capacitor *C* to a peak voltage *V*_c_; a series-blocking diode was necessary to prevent current backflow. Output across the capacitor provided continuous power to the single-bit counter. To count pulses from the Wiegand sensor, the application of a clock signal (CK) was necessary, as indicated in Figure 5a. The clock signal was generated using switch S, as shown in Figure 5b, and the on-off positions of the switch were controlled by the signal generated across the Wiegand sensor. A high resistance value was selected to prevent short circuiting as the switch S was turned on. In this way, the Wiegand sensor works as both a sensor and a power source.

## 4. Results and Discussion

The energy harvested by a Wiegand sensor is comparatively low (i.e., in the nJ region); however, due to advancements in low-power electronic devices, it seems possible to perform the required operations with the amount of energy generated. From an energy management point of view, the selection of the energy harvesting circuit, as well as the circuit parameters, is a crucial task.

### 4.1. Selection of the Optimal Capacitor Value

The filter capacitor value plays a vital role in the performance of the capacitor-based power supply scheme. The ripple voltage Δ*V_r_* across the filter capacitor is a function of the filter capacitance value, the input frequency, and the load current. For the complementary metal oxide semiconductor (CMOS) circuit, the permitted ripple voltage is approximately 10% [20]:

(5) $$\Delta V_{r}=V_{1}-V_{2}$$

where Δ*V_r_* is the peak-to-peak ripple, *V*_1_ is the peak value of input voltage, and *V*_2_ can be determined from the predefined allowable range of Δ*V_r_*. At a steady state, the energy harvested by the capacitor *E*_h_ is equal to the energy delivered to the load *E_load_*. For a period of *T*/2,

(6) $$E_{load}=\frac{1}{2}C\left(V_{1}^{2}-V_{2}^{2}\right)$$

The energy delivered to load *E_load_* in the period *T*/2 when the capacitor discharges is given as

(7) $$E_{load}=P_{o}\frac{T}{2}$$

From Equations (6) and (7), and since the rectified frequency is twice the input frequency, substituting *T* = 1/*2f* leads to a capacitor value of

(8) $$C=\frac{P_{o}}{2\left(V_{1}^{2}-V_{2}^{2}\right)f}$$

**Figure 6:**
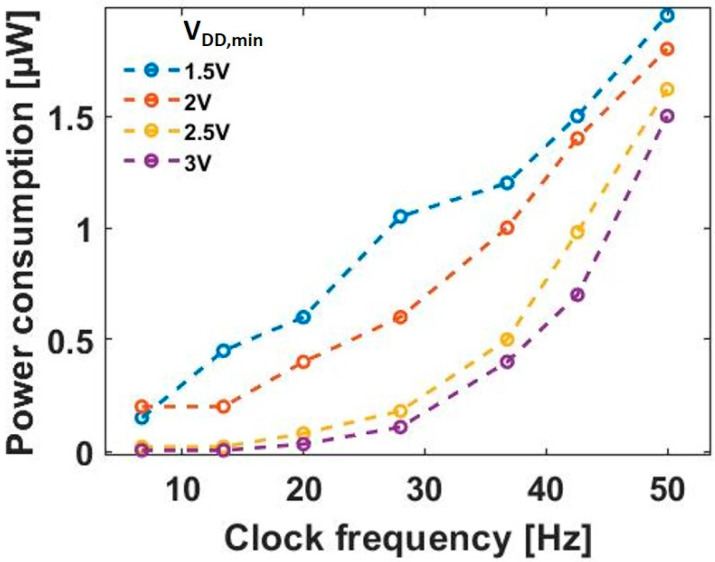
Power consumption of the D flip-flop (CD4031B) vs. clock frequency.

*P_o_* is the output power that is determined from the load requirements. CD4031B power consumption is a function of both the clock frequency and the supply voltage [21], assuming the load operates in the V_DD,min_ to V_DD,max_ voltage range; thus, it is measured by varying the clock frequency generated by the Wiegand sensor and by the external power supply at various V_DD,min_ values. The power consumption in the D flip-flop increases as a function of the clock frequency, as shown in Figure 6. However, as V_DD,min_ increases, the power consumption decreases comparatively. Therefore, the capacitor must be selected by considering the minimum power required to operate the D flip-flop at the lowest frequency. In this case, the output power *P_o_* required to drive the D flip-flop is about 0.1 µW for V_DD,min_ = 1.5 V. By substituting the value for minimum *f* (i.e., 1 Hz) into Equation (8), the optimal value of the capacitor *C* is determined to be 0.1 µF. The energy delivered to the load is ~38 nJ, which is in good agreement with the maximum energy generated of 130 nJ per pulse.

### 4.2. Comparing the Performance of an Active Rectifier and a Diode Bridge Rectifier

The performance of the full-wave rectifier also plays a key role in the efficiency of the proposed scheme. The performance of the two rectifier circuits, namely the active and diode bridge rectifiers, is compared in this section.

**Figure 7:**
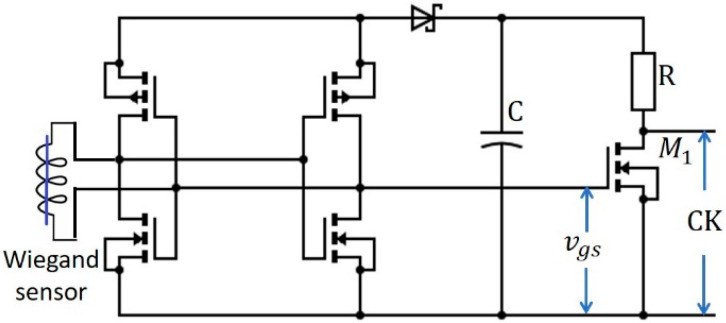
Proposed single-bit, self-powered counter with the active rectifier.

**Figure 8:**
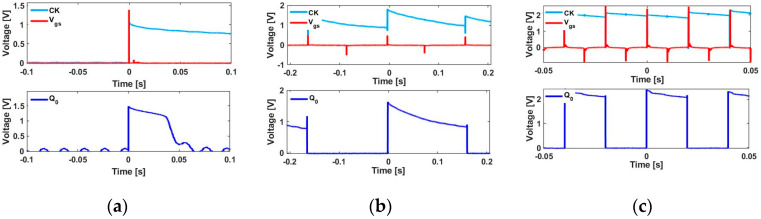
Experimental waveforms of the single-bit counter at various excitation frequencies using an active rectifier. Output *Q*_0_, CK (clock signal), and *V*_gs_ of single-bit D flip-flops (CD4013B) for (**a**) a single rotation and at excitation frequencies of (**b**) 5 and (**c**) 50 Hz.

As shown in Figure 7, the active rectifier [22] is implemented with two N-channel [23] and two P-channel [24] metal-oxide-semiconductor field-effect transistors (MOSFETs). The circuit parameters used for this implementation are listed in Table 1. For each positive peak, the capacitor *C* charges up to voltage *V*_c_, which supplies power to *M*_1_ and the D flip-flop. *M*_1_ is operated by the positive peak of the Wiegand sensor, as shown in the waveform of *V*_gs_. The output across *M*_1_ provides the clock signal to the flip-flop and is indicated as CK in the obtained waveforms. As the D flip-flop is a positive-edge type of triggered flip-flop, one square wave cycle is generated at *Q*_0_ for a single rotation, as shown in Figure 8a. In this study, the rotational frequency varied from 1 to 50 Hz. Figure 8b,c show the D flip-flop *Q*_0_ outputs for excitation frequencies of 5 and 50 Hz, respectively.

**Figure 9:**
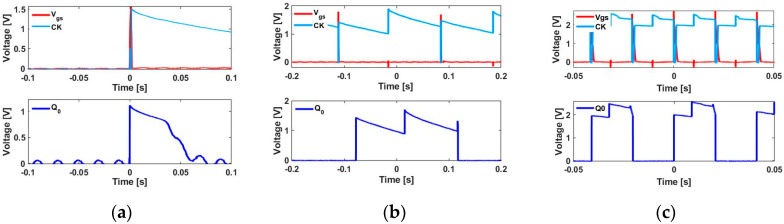
Experimental waveforms of the single-bit counter at various excitation frequencies using the diode bridge rectifier. The output *Q*_0_, clock signal (CK), and *V*_gs_ of the single-bit D flip-flop (CD4013B) for (**a**) a single rotation and for rotational frequencies of (**b**) 5 and (**c**) 50 Hz.

The active rectifier circuit in Figure 7 was replaced by a diode bridge rectifier, while all other circuit parameters were kept the same. A BAT85 Schottky barrier diode [25] was used by considering its low-forward-voltage drop. The results obtained for a single rotation and at rotational frequencies of 5 and 50 Hz are shown in Figure 9a–c. The results shown in Figure 9 are negatively impacted by the higher peak-to-peak ripple voltage when compared with the results reported in Figure 8.

### 4.3. Relationship between Output of the D Flip-Flop and Rotational Speed

All results reported in this study were obtained by inserting four magnetic reversals (N = 4); i.e., two cycles per revolution were generated across the output terminals of the Wiegand sensor that was rectified with a full-wave rectifier in four positive half cycles. As the MOSFET was triggered during the positive peak of the Wiegand sensor, and because the D flip-flop was triggered by a positive-edge pulse, the output frequency at *Q*_0_, which is denoted as *f_Q_*_0_, was half the rotational frequency. From Equation (2), the rotational speed is directly proportional to the rotational frequency and is given by

(9) $$S=60\;f_{Q_{0}}$$

In the developed scheme, a D flip-flop is powered continuously using a simple capacitor-based circuit. Furthermore, each Wiegand pulse powers a simple hardwired logic circuit, such as that of a nonvolatile ferroelectric random access memory (FRAM) element [26]. This logic performs a “+1” increment, stores the updated value, and finally powers the device to the OFF state using the energy harvested by the Wiegand sensor. In order to implement fully functional self-powered sensors with wireless transmission, one can charge the battery and transmit the stored data when the power required to drive an ultralow power transmitter is available, which will be realized in subsequent research.

## 5. Conclusions

This study developed a Wiegand sensor for use as an energy harvester for rotary applications. We showed that the Wiegand sensor can deliver energy in the presence of very-low-frequency external excitations, or even in a one-shot manner. In this study, considering the use of the Wiegand sensor in rotary applications enabled the establishment of a linear relationship between rotational motion, magnetic field reversal, and rotational frequency. This revealed that higher rotational frequencies can be achieved at lower speeds by increasing the number of field reversals. Therefore, higher power can be generated at a lower rotational speed. Secondly, the theoretical maximum available energy per pulse was derived analytically, and its value matched the experimentally obtained energy per pulse value of 130 nJ.

A single-bit, self-powered digital counter design with a D flip-flop was developed, in which the capacitor value is selected in such a way that continuous power is delivered to the D flip-flop. A minimum rotational frequency of 1 Hz was considered, and a single revolution or movement of the shaft equivalent to a speed of 30 rpm was counted using the developed scheme, with an energy ≈38 nJ delivered to the load. The performance of the active rectifier was compared with that of a diode-bridge rectifier. The results showed that the active rectifier generated an improved output in terms of the ripple voltage. The selection of the optimal value of the capacitor was also discussed. An output voltage of 1.5 V was obtained for a single revolution, and an even higher voltage as well as frequencies down to zero can be measured using complementary MOSFET with a lower *V*_th_ or by implementing other techniques.

Thus, the successful operation of the D flip-flop showed the strong potential of the Wiegand sensor for the advancement of IIoT considering its prominent applications in industrial turbine-type flowmeters in which the angular (rotational) velocity of the turbine rotor is directly proportional to the fluid velocity in the turbine.

## Figures and Tables

**Table 1:** Circuit parameters: single-bit digital counter using the active rectifier.

Component	Value
D flip-flop	CD4013B
N-channel MOSFET	2N7000
P-Channel MOSFET	ZVP442
Schottky diode	BAT85
Capacitance C	0.1 µF
Resistance R	200 kΩ
